# Learning processes and learning problems in German postgraduate medical education

**DOI:** 10.3205/zma001131

**Published:** 2017-11-15

**Authors:** Hendrik van den Bussche, Lea Krause-Solberg, Martin Scherer, Stine Ziegler

**Affiliations:** 1 University Medical Center Hamburg-Eppendorf, Institute of Primary Medical Care, Hambrug, Germany

**Keywords:** Postgraduate medical training, postgraduate medical education, residency, learning process, quality assurance, gender discrimination

## Abstract

**Objective:** In order to evaluate the quality of postgraduate medical education in Germany, we examined how the learning of theoretical and practical competencies is conceptualized and how the learning process takes place in real terms. The training conditions, as perceived by medical residents, are compared with the learning objectives, as stated by the Federal Chamber of Physicians in its regulation on postgraduate education. The analysis is based on the data of the “KarMed” study (abbreviation of "career progression and career breaks among physicians during postgraduate education"), a multicentre cohort study of medical school graduates from seven universities who arre followed until they receive their licenses as specialist physicians. The study was conducted by the Institute of Primary Medical Care of the University Medical Center Hamburg-Eppendorf starting in 2008.

**Methodology: **The KarMed study is based on annual, standardized surveys of the population described above. 48% responded in the initial survey (n=1012) and 85% or more in each of the following surveys. Descriptive statistics and logistical regressions were used for analysis. Gender-specific analyses were performed where possible in order to highlight the differences in the professional objectives and workloads between female and male physicians.

**Results: **The study shows that both the practical and the theoretical components of postgraduate medical education in Germany are insufficient. There is a lack of a curriculum with precise learning objectives and descriptions of the corresponding educational settings. In fact, the act of learning is identical to daily clinical work. There is no structuring of the work process with regard to learning; for example, documentation procedures and feedback discussions are prescribed but largely omitted. Evidence-based medicine is not a systematic part of the training, nor is the evaluation of residents’ progress. The summative final oral examination pretends that the necessary specialist competencies can be evaluated within 30 minutes.

Many factors indicate that female doctors, especially those with children, have fewer learning opportunities than male doctors.

**Conclusion:** The quality of postgraduate medical education in Germany has become inadequate, especially in an international comparison. The deficits are well known. The responsible institutions are called upon to implement sustainable reforms in the sense of postgraduate education that is structured according to educational principles and whose quality is assured.

## Zusammenfassung

**Zielsetzung: **Mit dem Ziel einer Bewertung der Weiterbildungsqualität von Ärztinnen und Ärzten in Weiterbildung (ÄiW) wurde untersucht, wie das Erlernen der theoretischen und praktischen Kompetenzen konzeptualisiert wurde und wie die Lernprozesse real ablaufen. Die durch die ÄiW wahrgenommenen Verhältnisse sollten den Lernanforderungen durch die Ärztekammern gegenübergestellt und diskutiert werden. Wesentliche Grundlage dieses Aufsatzes ist die KarMed-Studie („Karriereverläufe und Karrierebrüche bei Ärztinnen und Ärzten während der fachärztlichen Weiterbildung“), eine seit 2008/09 vom Institut für Allgemeinmedizin des Universitätsklinikums Hamburg-Eppendorf durchgeführte multizentrische Kohortenstudie von Absolventinnen und Absolventen des Medizinstudiums.

**Methodik: **Die KarMed-Studie beruht auf jährlichen standardisierten Befragungen der Absolventinnen und Absolventen des Medizinstudiums. Begonnen wurde im Jahr 2008/09. Beteiligt waren sieben medizinische Fakultäten (N=2012 in der Erstbefragung). Für die Auswertung wurden deskriptive Statistiken angewandt. Soweit möglich wurde nach Geschlechtern differenziert, um die bekannten Unterschiede in den Berufsvorstellungen und Belastungen zwischen beiden Geschlechtern beleuchten zu können.

**Ergebnisse: **Gezeigt wird, dass sowohl die praktischen als auch die theoretischen Komponenten der ärztlichen Weiterbildung in Deutschland unzureichend sind. Es fehlt ein Curriculum mit präzisen Lernzielen und darauf basierenden Beschreibungen der Lernsituationen, insbesondere solcher, die der Verbindung von Theorie und Praxis dienen. Die praktischen Lernbedingungen sind identisch mit der täglichen klinischen Versorgung unter Knappheitsbedingungen. Eine Strukturierung der Arbeitsabläufe im Hinblick auf die Qualifizierung fehlt, häufig auch die in der Weiterbildungsordnung vorgeschriebenen Dokumentationsvorgänge und Feedback-Gespräche. Die theoretische Fundierung der Weiterbildung weist insbesondere bezüglich der Vermittlung der Evidenzbasierten Medizin beträchtliche Defizite auf. Eine lernziel- und lernsettingbezogene Evaluation der Ärzte und Ärztinnen in Weiterbildung fehlt weitgehend. Die summative Abschlussprüfung behauptet, in dreißig Minuten die Frage klären zu können, ob die erforderlichen fachärztlichen Kompetenzen auch tatsächlich erworben wurden.

Viele Hinweise deuten darauf hin, dass Ärztinnen weniger Lernmöglichkeiten erhalten als Ärzte, obwohl sie eine stärkere Begleitung und Unterstützung erhalten sollten, insbesondere wenn sie Kinder haben.

**Schlussfolgerung:** Die Qualität der Weiterbildung von Ärztinnen und Ärzten in Deutschland ist unzureichend. Um im internationalen Vergleich von einer Außenseiterposition wegzukommen, müssten die beschriebenen Defizite behoben werden. Die Verantwortlichen werden dazu aufgerufen, nachhaltige Reformen im Sinne einer strukturierten und qualitätsgesicherten Weiterbildung umzusetzen.

## 1. Background

In all industrialized countries, the process of qualifying doctors is traditionally divided into two phases: undergraduate medical education (UGE) and, subsequently, postgraduate medical education (PGE), also called postgraduate training or vocational training. In Europe, both phases have a comparable real duration of approximately 6-7 years in most countries [1].

UGE in Germany is regulated by the Approbation for Physicians (“Approbation für Ärzte und Ärztinnen” [https://www.gesetze-im-internet.de/_appro_2002/BJNR240500002.html]), a directive issued by the Federal Ministry of Health with the consent of the Upper House of Parliament (“Bundesrat”), whereas PGE is based on regulations (“Weiterbildungsordnung” [http://www.aerztekammer-hamburg.org/wbo.html]) issued by the Regional Chambers of Physicians on the basis of a proposal (“Musterweiterbildungsordnung” [http://www.bundesaerztekammer.de/aerzte/aus-weiter-fortbildung/weiterbildung/muster-weiterbildungsordnung/]) developed under the aegis of the Federal Chamber of Physicians (“Bundesärztekammer”). 

The regional chambers’ responsibility for PGE is delegated by the governments of the regional states, which are supposed to control the chambers. In reality, PGE is completely steered by the chambers. While a plethora of political and scientific institutions and organizations comment on UGE, postgraduate education is almost completely out of public view. As a result, there have been only few studies and memoranda on PGE in Germany [2], [3], [4], [5], [6], [7], [8], [9], [10]. Questions concerning the future supply and demand of physicians play a role in the debates surrounding UGE, but this not the case with PGE. 

The analyses of PGE presented in this paper are largely based on the results of a multicentre prospective cohort study of graduates from seven medical schools (n=1012) conducted by the Institute of Primary Medical Care at the University Medical Center Hamburg-Eppendorf (“KarMed” study) since 2008. The KarMed study uses annual, standardised questionnaires to follow the graduates until they acquire their licenses as specialist physicians, including primary care specialists.

This paper examines, firstly, the question of how the theoretical and practical components of PGE are actually administered and acquired in the training hospitals. It also examines how the residents, on the one hand, and the Chambers of Physicians, on the other, assess the quality of the training. Special attention is given to gender differences.

## 2. Methods

Residents were followed using annually administered, standardised questionnaires during the period of their training from graduation to their licensure as a specialist physician. The results presented in this paper refer to the end of the fourth year of residency. In a few cases, results from previous KarMed surveys are also presented. Due to limitations of scope, reference must be made here to other KarMed publications [11], [12], [13], [14], [15], [16]. For the specific problems of PGE for primary care, we refer to separate publications [17], [18].

For further details on the methodology of the KarMed study see the paper by Ziegler et al. in this issue [16].

## 3. Results

### 3.1. Study cohort

For details on the KarMed cohort see Ziegler et al. in this issue [16].

#### 3.2. Learning conditions and learning outcomes 

Medical residents in Germany have the status of a physician employed by the hospital. They work full-time in the hospital without any protected time for research or educational activities. Training positions are not allocated; residents have to locate and apply for a position within the hospital labour market on their own. The employment contract does not contain any significant regulation regarding the educational entitlements and duties of the resident. There is no written agreement between the mentor and the resident describing the educational obligations of both sides.

The resident receives a full salary on the basis of the annual negotiations between the national trade union of physicians and the hospital trustee associations. Extra payment is provided for night and weekend duties. Current contracts vary between 38.5 and 48 hours per week, although the actual average shows a mean of 46 hours per week (night and weekend duties excluded).

As a result of this contractual status, residents are fully involved in the treatment of inpatients and outpatients in daily hospital care. Residents learn how to deal with a variety of patients and their conditions in all units of their discipline (in- and outpatient wards, intensive care, operating theatres etc.). The assignment to units and tasks is the responsibility of the chief physician of the department. Residents are assigned to units according to a simple scheme based on their presumed maturity (e.g. no intensive care unit in their first year). The extent to which the qualification of the individual resident is considered in these assignments may vary (see section 3.2.2). PGE is therefore characterized by learning on the job with very few organized learning events (e.g. seminars or courses) in either the training hospital or in the medical school (see section 3.2.3). 

##### 3.2.1. Training requirements in postgraduate educational regulations 

The regulations document issued by the Federal Chamber, as described in section 1, is the main regulatory instrument for PGE [http://www.bundesaerztekammer.de/aerzte/aus-weiter-fortbildung/weiterbildung/muster-weiterbildungsordnung/]. These regulations contain the following essential rules:

- Minimum number of months/years in the main and adjacent discipline and/or health care facilities (primarily hospitals) needed for graduation.
- Minimum numbers of procedures to be performed (operations, diagnostic interventions) contained in so-called "catalogues".
- The obligation to document the procedures described above annually in a "logbook", to be signed by the mentor of the resident. This is regularly the chief physician of the department.
- Annual interviews between mentor and resident on the basis of the logbooks, in which the state of progress is to be judged by both. Existing deficits must be noted. The minutes of these interviews must be signed by both and attached to the application for admission to the final examination (see below).
- The training hospital must deliver a so-called "structured training plan" to the resident at the start of the training. This usually includes little more than the pathway through the department units in the individual years of training.
- At the end of PGE, the mentor has to issue a certificate to the resident, in which the experience and skills of the resident are described and it is stated that the candidate is fully qualified for the specialist certificate.

##### 3.2.2. The state of practical training

As stated above, PGE consists almost exclusively of daily work in the hospital. As a consequence, access to interventional activities (surgical procedures, endoscopies etc.) is also unregulated. The decision that a resident may participate in such activities is made primarily according to the daily requirements of the department and the availability of the medical workforce, with little basis in the learning interests of the residents.

The lack of regulations concerning access to learning settings and daily work experience leads to competition among nearly all physicians in a department [19], [20]. Younger physicians would like to quickly participate in the easier procedures listed in the catalogues in order to end up with a full portfolio at the end of their PGE. However, residents at the end of their training do not always feel qualified enough to practice independently. In addition, they also want to gain experience with more complex interventions. This also applies to the licenced specialists who remain in the hospital in order to qualify for a position as deputy director (“Oberarzt”), in order, for example, to be able to apply for a chief physician position in a district hospital later on. The less attractive tasks, such as ward work or outpatient consultations, are assigned to the younger residents. In this open competition, success is largely dependent on the presence and competiveness of the individual residents (see Ziegler et al. in this issue on the differences between male and female physicians; [16]).

Attitudes of the residents in the KarMed study towards their practical learning conditions after four years of PGE are shown in Table 1 (Tab. 1). The attitudes were expressed on a five-point Likert scale (minimum agreement=1, maximum agreement=5, mean of scale=3).

The items related to mentor and team (1-6) reveal that central competencies of the PGE and central educational activities (e.g. receiving feedback) received somewhat moderate scores. Negative estimations were given by female residents regarding their learning to take responsibility in patient care (row 1) and their reception of constructive feedback on the quality of their practical skills (row 5). In both cases, male physicians appreciate their training significantly more. 

Those items in Table 1 (Tab. 1) referring to the organization of practical training (items 7-10) show further significant deficits. Only 19% of the respondents agreed that there was a structured training plan that was also respected. Comparable negative results are shown in eight other studies [http://www.bundesaerztekammer.de/fileadmin/user_upload/downloads/EVA_Bundesrapport_final_16042010.pdf], [http://www.bundesaerztekammer.de/fileadmin/user_upload/downloads/BAeK_Ground4.pdf], [http://www.marburger-bund.de/projekte/mitgliederbefragung/2014], [21], [22], [23], [24], [25]. Likewise, more than half (58%) of the respondents disagreed with the statement that "the interventions and operations to be documented for the final examination are regularly registered (e.g. in a logbook)”, and only 25% agreed explicitly with this statement. Comparable sizes were obtained in other surveys [http://www.marburger-bund.de/projekte/mitgliederbefragung/2014], [23]. In addition, 44% of the respondents answered that "leading physicians regularly conduct interviews with the residents". Other surveys yield comparable results [21], [23], [26]. When asked about mentoring, only 31% of the residents declared that they also received instruction from the chief physician of the department, who is usually the official mentor. Female physicians (29%) had a significantly (p=0.034) more negative opinion than males (34%). Further critical results on the real role of the PGE mentors are also found in other studies [21], [22], [27].

In this respect, critical results in other studies on the conditions and outcomes of PGE were found for anaesthesiology [23], [24], surgery [25], [26], [27] and internal medicine [21], [26].

##### 3.2.3. The missing theoretical foundation of postgraduate medical education

Every effective practical learning process needs a theoretical foundation. The term "theoretical" in this study refers to all structured teaching and learning events, inside and outside the hospital, which aim at systematically improving the knowledge, skills, and attitudes of the residents. In this sense, theoretical learning includes the demonstration and exercise of practical skills, for example in a simulation session. As stated before (see section1), it is, however, typical for postgraduate medical education in Germany that this theoretical foundation is almost completely absent during the entire PGE process. A curriculum describing the learning objectives, the educational settings, and the methods for evaluating the residents’ progress usually does not exist. See section 4 for a more specific discussion of the causes and consequences of this situation.

The KarMed survey also revealed considerable weaknesses in the theoretical underpinning of PGE programs, especially in hospitals with fewer than 800 beds. Just under half (females 44%, males 47%) of the respondents stated that they received feedback on the state of their theoretical knowledge, and only one quarter (24%) reported to be occasionally instructed to improve their knowledge. Female physicians reported significantly less frequently (20%) than males (34%) to have learned to apply the results of scientific studies (p=0.029). As a result, the mean values of the responses from the residents regarding the learning conditions for theory (Likert scale, minimum=1, maximum=5, scale mean=3) were very negative (see Table 2 (Tab. 2)).

Since the chambers assign so little importance to the theoretical component, there are few surveys in which the theoretical component of PGE is investigated (see section 3.2.5).

The contribution of the hospitals to the theoretical foundation of PGE is, in effect, restricted to short, in-house conferences that take place, on average, once a month. The quality of these sessions depends to a large extent on the academic and didactic qualifications of the individual mentors, and is thus largely unpredictable. Interviewees in the KarMed survey reported an average of one hour per week of in-house training in the first year of training (see Table 3 (Tab. 3)). According to the German Professional Association of Surgeons in 2009, 39% of surgical residents reported participating in a maximum of four sessions lasting one to two hours per year [25]. Participants in the KarMed study reported the following figures for different theory-related learning methods in the first year of residency (see Table 3 (Tab. 3)):

Even generously assuming that each resident may employ all of the four learning methods presented above, the result would be a theory-related volume of four hours per week, during the year with arguably the largest demands for learning. Also, when the residents were asked which journals they most frequently read, the classical scientific journals of medicine (British Medical Journal, New England Journal of Medicine, Lancet, etc.) were hardly mentioned.

##### 3.2.4. The global appraisal of PGE by residents

In view of the results presented so far, it is not surprising that the majority of the studies assessing the opinions of residents on their PGE have shown negative opinions. In the KarMed study, less than half of the respondents (42%) agreed with the statement that "providing a good education is an important goal of our department / hospital", and only half of the respondents (52%) approved the statement, "I would recommend my department/clinic to younger colleagues with regard to the quality of their training". Other studies – none of which, unfortunately, differed according to gender – came to comparable or often even more critical results [http://www.marburger-bund.de/projekte/mitgliederbefragung/2014], [http://www.yumpu.com/de/document/view/7436730/online-blitzumfrage-der-dgim-zeigt-erhebliche-defizite-im-bereich], [22], [25], [27], [28]. In conclusion, according to these studies, between 25% and 60% of the residents showed a positive attitude towards their PGE. 

##### 3.2.5. The minimization of PGE deficits by the chambers

It should be noted positively that the Federal Chamber investigated the opinions of the residents on the quality of PGE by means of nationwide, standardized surveys in 2009 and 2011 [http://www.bundesaerztekammer.de/fileadmin/user_upload/downloads/EVA_Bundesrapport_final_16042010.pdf], [http://www.bundesaerztekammer.de/fileadmin/user_upload/downloads/BAeK_Ground4.pdf]. The Federal Chamber rated the overall results for 2009 as a solid "B-", elsewhere as "good", and the comparable results in 2011 as "relatively good". According to the Federal Chamber, the overall average of 2.54 on a six-point scale (score scale 1=very positive, 6=very negative; scale middle=3.5) "reflects the basic satisfaction (of the residents) with the situation of PGE in Germany”. It was overlooked that results in the positive range of the scale (1-3.4) were often due to the very positive results of PGE for general practice, whereas the hospital disciplines often received negative ratings. This was especially the case for the application of evidence based medicine (EBM). Here, significant negative values (>3.5) were found for all major clinical disciplines (e.g. 4.0 for obstetrics and gynaecology, as well as for paediatrics).

## 4. Discussion: The causes of the misery

### 4.1. Non-intervention as a management principle

The data in section 3.2.2 show that the official instruments for documentation and evaluation of the residents’ progress are not regularly utilised by the mentors. Since these logbook documents must be submitted with the signatures of the mentor(s) for admission to the final examination, these interventions seem to be documented after the fact, at the end of the program, rather than annually, in the course of the training. One should bear in mind that these documents constitute official administrative records under public law. The chambers do not publicly criticize these practices or take action against them. 

Also, there are no criteria for the “structured training scheme” to be delivered to the residents at onset. In practice, these schemes consist of a short timetable describing the order in which the resident is to pass through the units of the department (for example, "intensive care unit in the 3rd semester"). The fact that such a scheme is often referred to as the "curriculum" shows the chambers’ deficits in educational matters. 

#### 4.2. Gender-specific inequalities

The conditions of postgraduate education, as laid down in the Federal Chamber regulations and put into practice by the hospitals, can be regarded as man-made directives for men who have a female partner at home to support them. The KarMed study provides plentiful evidence that female physicians have to cope with greater problems than their male counterparts from the beginning of postgraduate training. A female physician who has no children yet is considered to be a "pregnancy risk", something that is often openly addressed during the application process, although it is legally forbidden [12], [15]. As a consequence, chief physicians give less appreciation to applications from female doctors. "Climatic" forms of discrimination by male physicians in daily work are often reported. The professional problems of female doctors increase substantially when they have children [12], [13], [16]. For the most part, having children results in a transition to part-time work and a lower availability in the hospital, and, subsequently, to fewer career opportunities, an extension of the length of training, and, often, an interruption or discontinuation of the residency. This is associated with differences in endorsement and promotion between male and female residents by mentors, as described in sections 2.3.2 and 2.3.3. Female residents currently constitute two-thirds of all trainees, twice the percentage of 20 years ago [29]. There is an urgent need to develop residency concepts more adapted to the private conditions of female residents and to promote professional cultures that value the work of female physicians who have children. Otherwise, the future supply of physicians in certain areas of medicine, such as the surgical disciplines, may become insufficient in German hospitals.

#### 4.3. Evaluation deficits

It is implicitly assumed in German PGE that a several years of working in a hospital, within an otherwise unstructured teaching and learning environment, somehow automatically guarantees the necessary competencies of a specialist physician. However, a number of years employed in a hospital need not necessarily coincide with the required qualification. The only examination in the course of residency is structured to take thirty minutes and is not considered to be an examination but rather a “discussion among colleagues” (“Fachgespräch”). A 30-minute conversation appears to be an audacious approach to validly examining the competencies of a young specialist. The same problem arises in the course of training: How can the mentor intervene and promote the professional development of the resident if his or her knowledge, skills, and attitudes are not pursued in a systematic manner? A variety of knowledge and skill tests have been developed in other countries, but these are neither recognized nor applied in German PGE [30], [31], [32], [33], [http://www.gmc-uk.org/education/27394.asp].

#### 4.4. "Ultimately", postgraduate training is not education

All assistant positions are fully accounted for in the hospitals’ budgets. Time equivalents for teaching and/or learning are not provided, and rewards for quality teaching and learning do not exist. The specifications for training are stated as minimal requirements, thus shifting the obligation to fulfil the requirements exclusively to the trainee. As a result, the core elements of any structured educational process – the definition of teaching and learning objectives based on professionally required competency profiles, the description of corresponding learning settings, and the construction of valid progress and outcome evaluation procedures, all three elements linked to each other in a "constructive alignment" [32], [34] – are completely lacking in the chamber regulations.

The deficits of postgraduate education due to the primacy of hospital work are well known. Jörg Ansorg, chief manager of the Federation of German Surgeons, described the problem as follows: "It is less the regulation framework that is insufficient, and more the faulty implementation in the hospitals and the non-existing sanctions by the chambers" [35]. In 2012, prominent professors in anaesthesiology concluded that "all the available results (indicate) [...] that PGE in Germany does not meet the high national demands for quality of care, patient safety, and the future viability of medical practice" [24].

The interest in an effective medical workforce is supported by what is most likely a mutual disinterest in theory and examinations. This is well illustrated by the low value placed on evidence based medicine (EBM) in actual postgraduate training, as described in the next section

#### 4.5. The theory deficit: “Eminence” based medicine?

Postgraduate training takes place in a rapidly developing world of medical knowledge, which necessitates reflection and self-evaluation of one’s own practice in order to assure both patient safety and the optimal allocation of resources. Evidence based medicine demands clinical action to be based on scientifically verified diagnostic and treatment concepts. The opinion became increasingly widespread that medical decisions based on traditions or the opinions of the medical leaders - ironically referred to as "eminence based medicine” – should be replaced by empirical evidence to the greatest extent possible.

The EBM principle is the opposite of learning by imitation and the transmission of local treatment concepts and procedures to the resident. The aim of EBM, however, is not to replace clinical experience with rigid guidelines [35], but to combine the application of established knowledge with the best clinical experience for the benefit of the individual patient [36], [37]. Central instruments of a future PGE that are based on an approach to EBM that is understood in this manner include the availability of information systems (such as guideline banks, Cochrane review database), the ability to retrieve such information, the routines to use them in daily care, and the development of adequate learning settings (such as interdisciplinary case conferences, mortality conferences, error reporting systems and the “good old” autopsy rounds). The performance and critical discussion of literature reviews, clinical studies, and individual clinical cases in group sessions and seminars [38], [http://www.bundesaerztekammer.de/fileadmin/user_upload/downloads/CurrEBM.pdf] are essential for residents to acquire the corresponding skills. The reality of German PGE is the contrary: The absence of EBM in German PGE was the most prominent complaint expressed by residents in the evaluative surveys of the Federal Chamber in 2009 and 2011 [http://www.bundesaerztekammer.de/fileadmin/user_upload/downloads/EVA_Bundesrapport_final_16042010.pdf], [http://www.bundesaerztekammer.de/fileadmin/user_upload/downloads/BAeK_Ground4.pdf]. Furthermore, a recent survey among all institutions responsible for UGE or PGE showed an “undersupply of training opportunities in EBM [...] in the all German-speaking countries, in particular in postgraduate and continuing education" [39].

## 5. Conclusion

The problems of PGE described here can only be solved by a better integration of undergraduate and postgraduate medical education. Such integration would view UGE and PGE as two phases of a single qualification process, and each would be provided with adequate proportions of theory and practice on the basis of the competency profiles of the individual medical disciplines [40].

In an international comparison, the German concept of PGE appears to be far behind modern OECD countries in PGE matters. The possibility that graduates of German PGE might face recognition difficulties in other countries in the future cannot be ruled out. The same applies to the recognition of training institutions according to the criteria of foreign agencies [https://www.acgme.org/acgmeweb/].

The results of this study show the need for further systematic investigations on the quality of PGE in Germany. At the same time, the results presented here should already be enough to stimulate the responsible institutions – the Chambers of Physicians, the specialist associations, and even the health ministries – to achieve sustainable reforms in German PGE in the sense of a truly structured education that is clearly founded in evidence based medicine and that implements measures for quality assurance.

Furthermore, in spite of its complexity, we see a need for international comparisons of process and outcome quality of different PGT concepts. 

## Funding

The KarMed-study was supported by the Federal Ministry of Education and Research and the European Social Fund in the years 2008 till 2014 (grant numbers 01FP0803 and 01FP0804). Since 2015 the study is supported by the National Association of Statutory Health Insurance Physicians (“Kassenärztliche Bundesvereinigung“). 

## Competing interests

The authors declare that they have no competing interests. 

## Figures and Tables

**Table 1:**
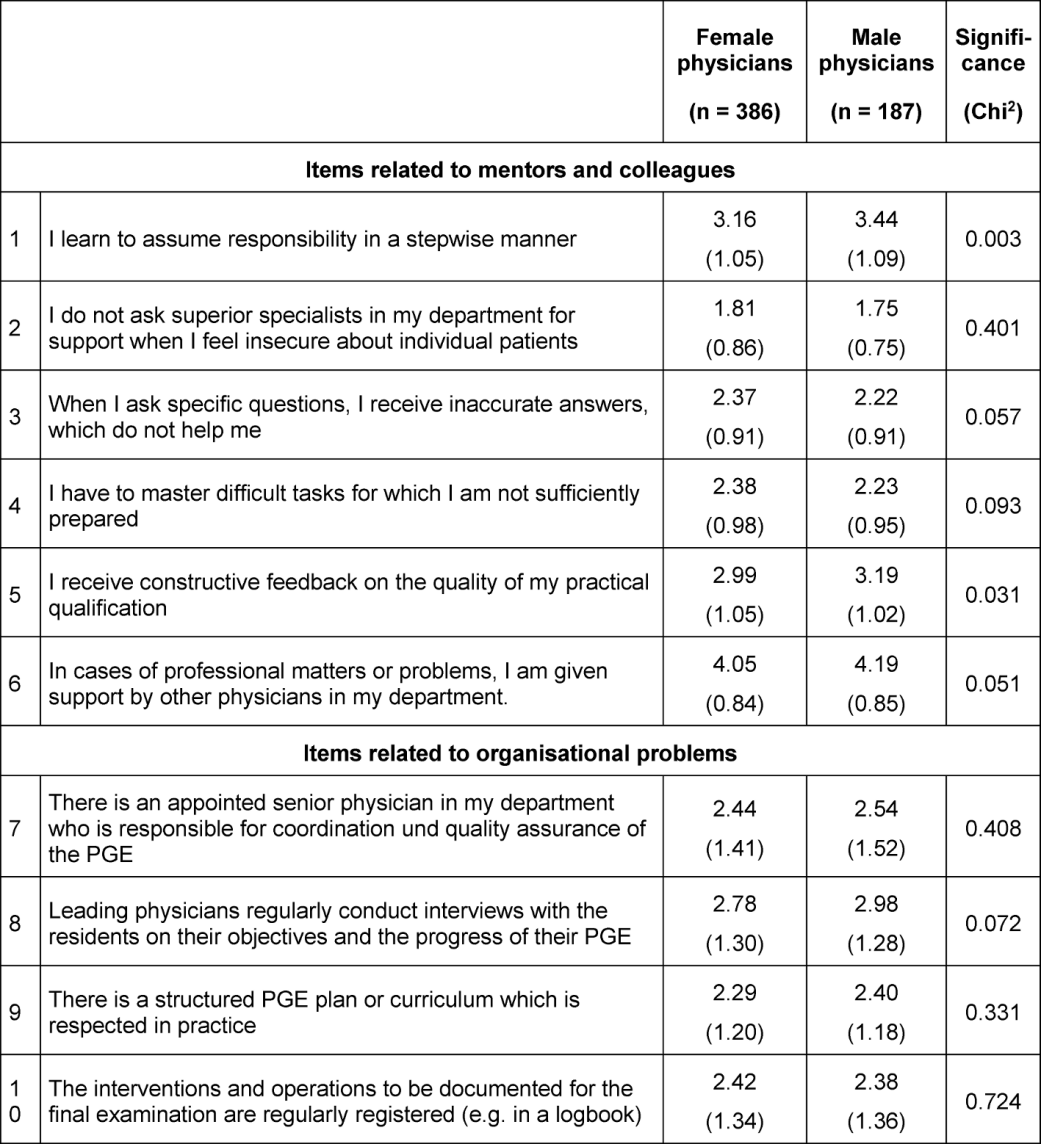
Means and standard deviations (in brackets) of the attitudes of German medical residents with regard to their practical learning conditions (N=583)

**Table 2:**
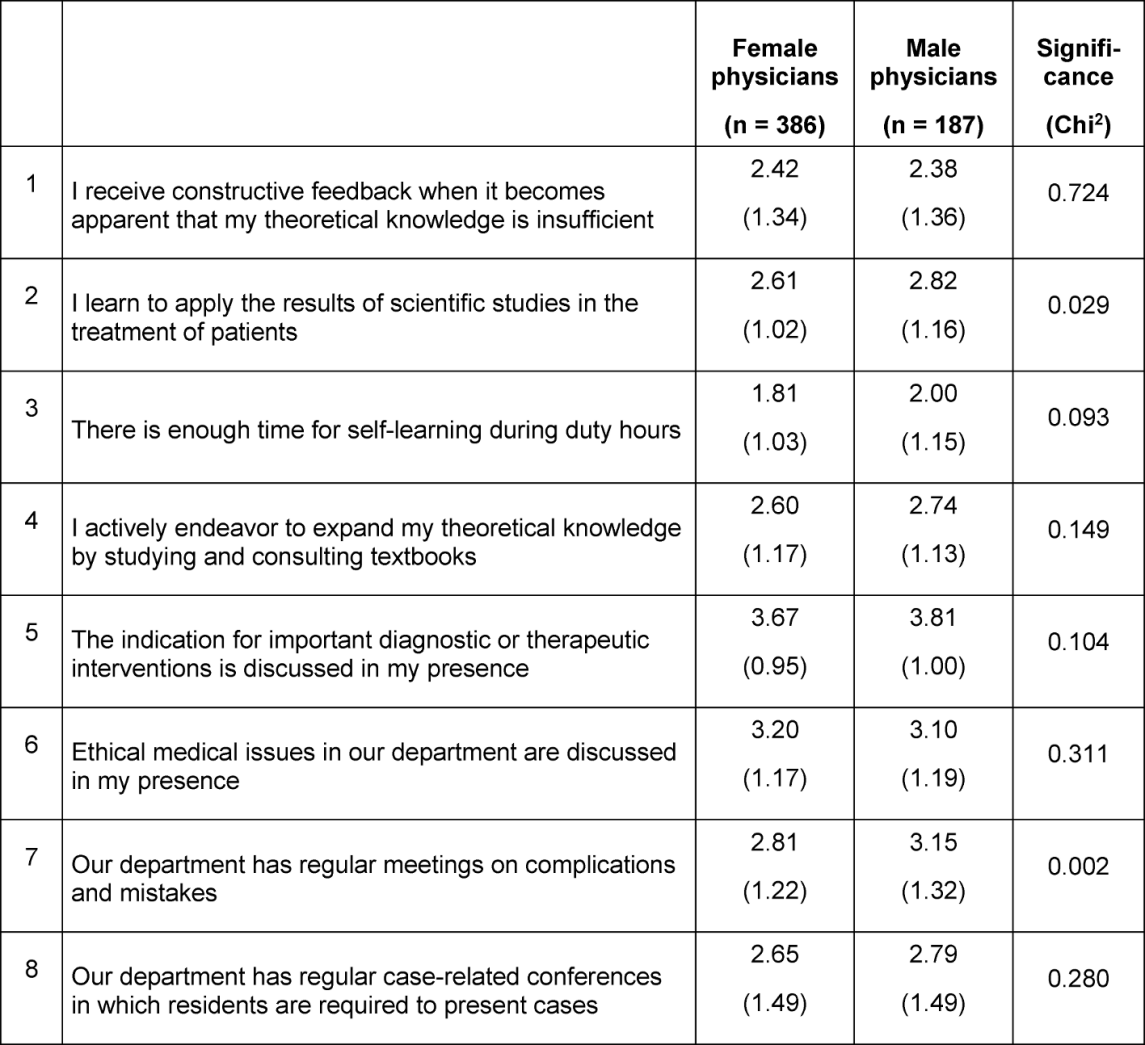
Means (standard deviations in brackets) of residents’ opinions on learning conditions for theory after four years of PGE (N=583)

**Table 3:**
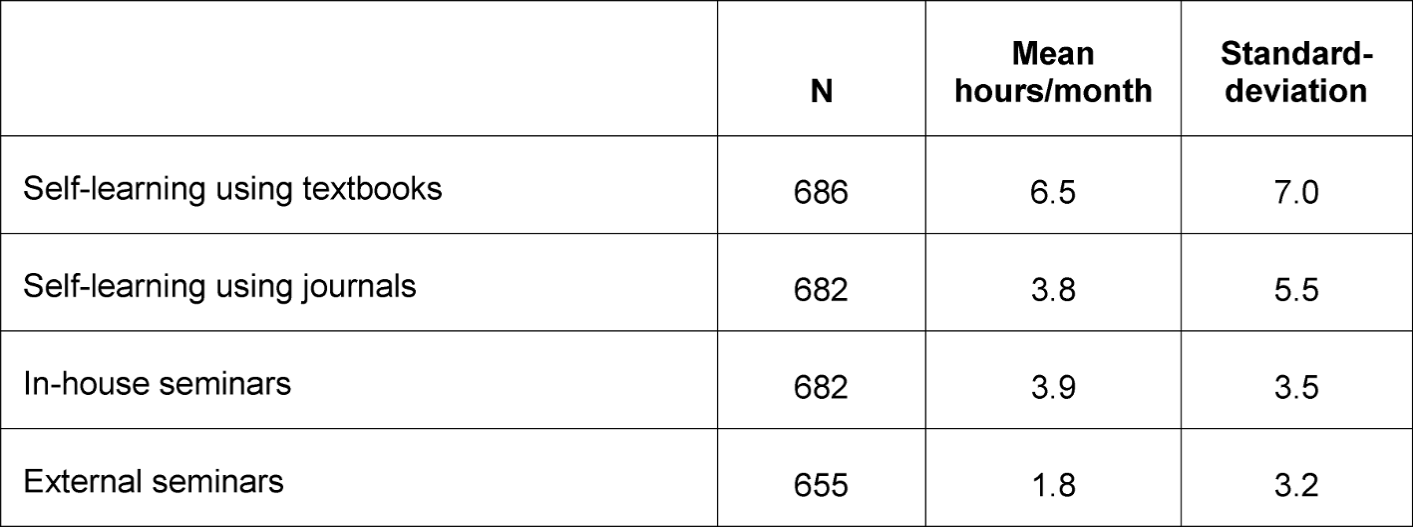
Amount of residents’ theory-related learning during the first year of residency in hours per month
